# Truncated protein tyrosine phosphatase receptor type O suppresses AKT signaling through IQ motif containing GTPase activating protein 1 and confers sensitivity to bortezomib in multiple myeloma

**DOI:** 10.18632/oncotarget.23017

**Published:** 2017-12-07

**Authors:** Hua Wang, Veerabhadran Baladandayuthapani, Zhiqiang Wang, Heather Lin, Zuzana Berkova, Richard E. Davis, Lin Yang, Robert Z. Orlowski

**Affiliations:** ^1^ Department of Lymphoma/Myeloma, The University of Texas MD Anderson Cancer Center, Houston, TX, USA; ^2^ Department of Biostatistics, The University of Texas MD Anderson Cancer Center, Houston, TX, USA; ^3^ Cyrus Tang Hematology Center, Soochow University, Suzhou, China; ^4^ Department of Experimental Therapeutics, The University of Texas MD Anderson Cancer Center, Houston, TX, USA

**Keywords:** multiple myeloma, drug resistance, AKT, GTPase activating protein 1, tyrosine phosphatase

## Abstract

Proteasome inhibitors are an important part of our chemotherapeutic armamentarium against multiple myeloma, but the vast majority of patients eventually develop drug-resistant disease through incompletely understood mechanisms. Comparison of gene expression profiles (GEPs) of bortezomib-resistant (BR) myeloma cell lines with their drug-naïve counterparts revealed decreased expression of truncated Protein tyrosine phosphatase receptor-type O (*PTPROt*) in BR cells. Over-expression of wild-type PTPROt in drug-naïve and BR cells reduced myeloma cell proliferation, induced apoptosis, and sensitized cells to bortezomib and to alkylating agents. PTPROt expression reduced AKT phosphorylation and activity, and sensitized to pharmacologic AKT pathway inhibitors, but this was not the case for a substrate-trapping catalytic domain-inactivating mutant. Co-immunoprecipitation and mass spectrometry studies identified IQ motif containing GTPase activating protein 1 (IQGAP1) as a PTPROt binding partner, and PTPROt reduced tyrosine phosphorylation of IQGAP1, providing a link to AKT activity. Analysis of clinically annotated GEP databases identified high *PTPROt* expression as being related to an increased likelihood of achieving complete remission with bortezomib therapy, while low expression was linked to a greater likelihood of disease progression. Finally, high *PTPROt* expression associated with prolonged median overall survival in patients receiving bortezomib-based therapy in the front-line or relapsed and/or refractory settings. Taken together, these data identify *PTPROt* suppression as a novel mechanism of myeloma resistance to bortezomib in myeloma cell lines, and also support the possibility that *PTPROt* expression could be used as a biomarker to predict outcomes with bortezomib, and by which to select patients for therapy with AKT inhibitors.

## INTRODUCTION

Multiple myeloma is a malignancy characterized by proliferation of clonal plasma cells and is the second most commonly diagnosed hematologic malignancy [1]. Due to an aging population, myeloma cases will grow almost 60% between 2010 and 2030, ranking it third among all cancers in the rate of increase during this period [2]. Recent advances in the treatment of myeloma, including the introduction of several categories of novel agents, have contributed to a doubling of the median overall survival in myeloma patients [3, 4]. Among the most important of these classes are drugs that inhibit the proteasome [5, 6] such as bortezomib, and thereby interfere with intracellular regulated proteolysis, which occurs predominantly through the ubiquitin-proteasome pathway [7]. Protein turnover capacity is reduced during plasma cell differentiation, which increases proteasome load relative to capacity, and thereby induces cellular stress [8], which may explain the exquisite sensitivity of this tumor type to bortezomib, which upsets this balance further [9].

Despite the strong efficacy of proteasome inhibitors in the treatment of myeloma patients in the front-line, relapsed, refractory, and other settings, the majority of patients develop resistant disease [10] through mechanisms that are as yet incompletely understood. Early studies based on pre-clinical models described a role in resistance for bortezomib binding pocket mutations in the β5 proteasome subunit [11–13]. However, these mutations were later found to be absent from primary patient samples [14, 15], suggesting that they are not physiologically relevant. A more recent study utilizing both pre-clinical models and clinical data demonstrated the emergence of plasmablasts with reduced immunoglobulin production as another potential mechanism [16]. This would predict that all bortezomib-resistant patients should have non-secretory myeloma, and while this can be seen [17], it occurs in only a small fraction of patients. Thus, other mechanisms must contribute to acquired, or secondary resistance, as well as to primary, or de novo bortezomib resistance, which is demonstrated by the fact that the response rate in proteasome inhibitor-naive patients in the relapsed/refractory setting is under 50% [18, 19].

To better understand possible mechanisms of resistance, our group developed bortezomib-resistant human myeloma cell lines which, like primary samples, proved to have no β5 proteasome subunit mutations [20, 21]. In the current work, we used gene expression profiling (GEP) to identify down-regulation of Protein tyrosine phosphatase receptor-type O truncated (*PTPROt*) as being associated with bortezomib resistance. Conversely, over-expression of PTPROt induced cell cycle arrest and apoptosis, and also enhanced sensitivity to bortezomib. Mechanistic studies showed that PTPROt expression suppressed signaling through Protein kinase B/AKT, and this seemed to occur through an interaction with IQ motif containing GTPase activating protein 1 (IQGAP1). Importantly, higher *PTPROt* expression levels were associated with a greater likelihood of achieving a complete remission to single-agent bortezomib therapy, and with a longer survival after bortezomib therapy in the clinic. These data suggest that *PTPROt* expression levels could be used to predict which patients could most benefit from bortezomib-based therapy, and point to strategies targeting AKT signaling in patients with low *PTPROt* expression as a possible mechanism to overcome resistance.

## RESULTS

### PTPROt and bortezomib resistance

**Figure 1:**
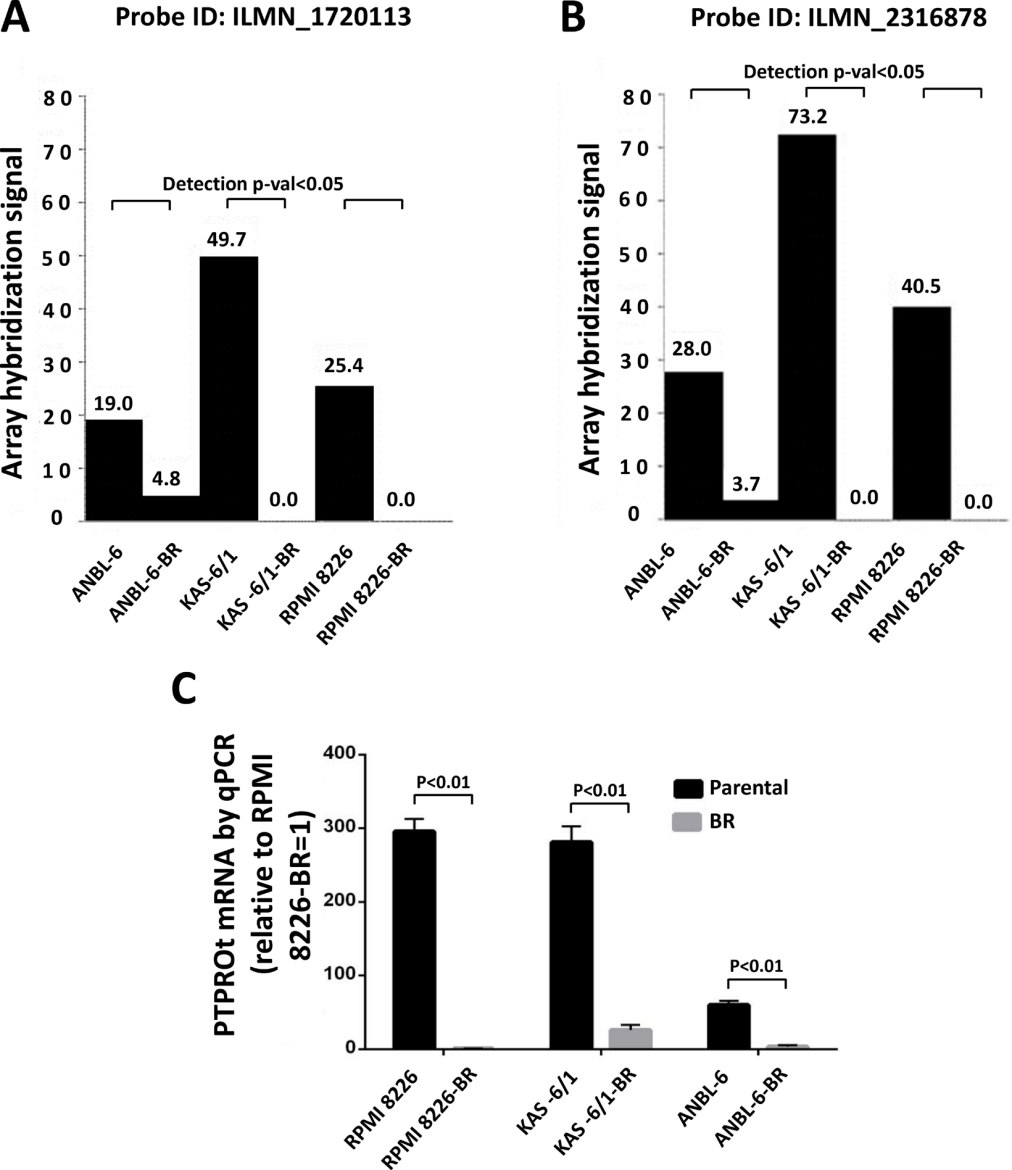
*PTPROt* expression in bortezomib-resistant and drug-naïve cell lines Gene expression profiling data from Illumina microarrays showing *PTPROt* expression in bortezomib-resistant (BR) cell lines and their drug-naïve counterparts at *PTPRO* probe (**A**) ILMN_1720113 and (**B**) ILMN_23168783. (**C**) Quantitative real-time PCR analysis of these same cell lines is shown with data normalized to the signal in RPMI 8226-BR cells, which were arbitrarily set to 1.0. Error bars indicated standard deviation, and the differences between the BR cells and their controls were all significant (*P* < 0.01 by the Students *t*-test).

To better understand mechanisms responsible for bortezomib resistance (BR), we analyzed GEP data comparing drug-naïve ANBL-6, KAS-6/1, and RPMI 8226 myeloma cell lines and their BR counterparts. In particular, we searched for genes that would be significantly either up- or down-regulated consistently in all three model systems. *PTPROt* met these criteria in that ANBL-6, KAS-6/1, and RPMI 8226 BR cells had lower *PTPROt* expression at both the ILMN_1720113 (Figure 1A) and ILMN_23168783 (Figure 1B) probes for this gene on the Illumina microarray. To confirm these microarray data, we performed quantitative RT-PCR on RNA independently extracted from the same paired cell lines. These studies also showed reduced expression of *PTPROt* in the BR cells, which ranged from a 3- to 300-fold reduction (Figure 1C).

### Impact of PTPROt on proliferation and apoptosis

**Figure 2:**
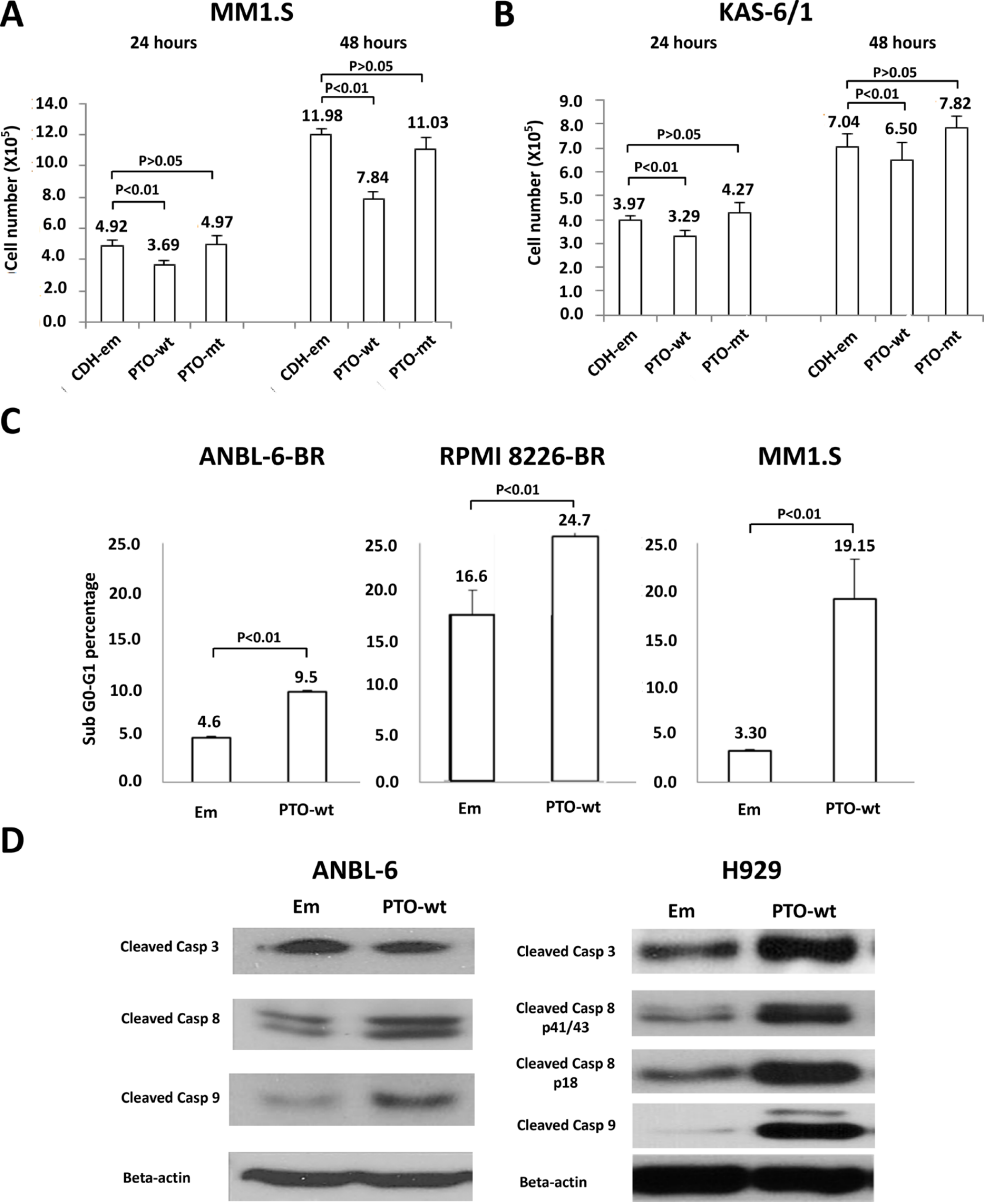
Expression of *PTPROt* and cell proliferation and apoptosis Myeloma cell lines were transduced with Lentiviruses expressing wild-type *PTPRPOt* (PTO-wt), a substrate-trapping catalytic domain-inactivating mutant (PTO-mt), or an empty vector control (CDH-em). Cells expressing the constructs were selected by flow cytometry for green fluorescent protein expression, and then seeded into 12-well plates at a density of 3 × 10^5^ cells/well and cultured. The cell number was counted in (**A**) MM1.S and (**B**) KAS-6/1 cells at 24 (left set of bars) and 48 (right set of bars) hours, with all experiments performed in triplicate. Error bars indicate standard deviation, and the differences between the wt cells and their controls were all significant (*P* < 0.01 by the students *t*-test). (**C**) ANBL-6-BR (left panel), RPMI 8226 BR (middle panel), and MM1.S cells with either an empty vector (Em) or wt *PTPROt* were evaluated for cell death by propidium iodide staining and flow cytometry. Bar graphs show the percentage of cells in the apoptotic sub-G_0_/G_1_ fraction, error bars indicate standard deviation, and the differences between the wt cells and their controls were all significant (*P* < 0.01 by the students *t*-test). (**D**) ANBL-6 (left panel) and H929 (right panel) cells prepared as above were lysed and extracts were subjected to Western blotting looking for the abundance of cleaved Caspases (Casp) 3, 8, and 9, with β-actin as a loading control.

PTPROt has been described to play roles in B-cell receptor signaling and proliferation [22, 23], but it has not been linked to the pathobiology of multiple myeloma. To better understand its potential role in plasma cells, we next over-expressed either wild-type (wt) PTPROt or a substrate trapping phosphatase-dead domain mutant (DC-mt), and compared their effects to those of an empty vector. Studies of cell proliferation showed that over-expression of wt PTPROt, but not the kinase-dead DC mutant, significantly slowed proliferation of drug-naïve MM1.S (Figure 2A) and KAS-6/1 (Figure 2B) myeloma cells versus controls. Since especially low *PTPROt* expression was seen in the bortezomib-resistant cells, we then over-expressed it in ANBL-6-BR and RPMI 8226-BR cells, and also found reduced proliferation (not shown). Cell cycle analysis showed that these cells, as well as drug-naïve MM1.S cells over-expressing wt PTPROt, had an increase in the sub-G_0_/G_1_ fraction (Figure 2C). Consistent with the possibility that there was at least some induction of programmed cell death, forced PTPROt expression increased levels of cleaved Caspases in ANBL-6 and H929 myeloma cells (Figure 2D).

### PTPROt acts as a chemosensitizer

**Figure 3:**
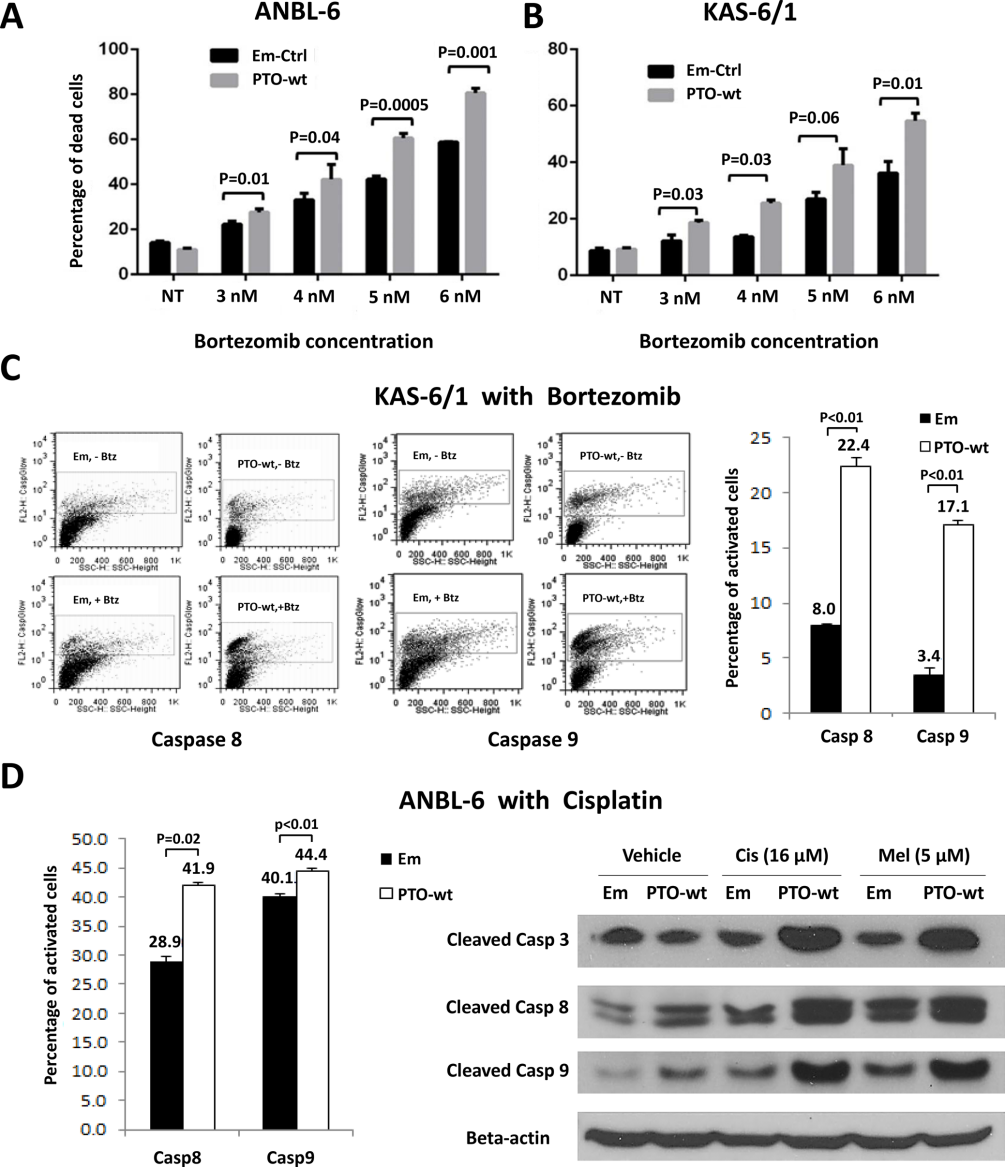
*PTPROt* and chemosensitivity in multiple myeloma (**A**) ANBL-6 or (**B**) KAS-6/1 cells transduced with an empty vector (Em-Ctrl) or one expressing wt *PTPROt* (PTO-wt) were exposed to either vehicle or the indicated concentrations of bortezomib for 24 hours. Programed cell death was examined by propidium iodide staining and flow cytometry as described above. Bar graphs show the percentage of cells with a sub-G_0_/G_1_ DNA content, and statistical significance data are provided in the panel. (**C**) KAS-6/1 cells with either an empty vector or wild-type *PTPROt* were exposed to vehicle or bortezomib and analyzed for Caspase 8 (left panel) or Caspase 9 (middle panel) cleavage using fluorogenic substrates and flow cytometry. (**D**) ANBL-6 cells with an empty vector or wt *PTPROt* were exposed to either cisplatin (16 μM) or melphalan (5 μM) and analyzed for cell death by flow cytometry (left panel) for Caspase 8 or 9 cleavage or by Western blotting (right panel).

In that PTPROt by itself induced apoptosis, it seemed possible that it could also enhance sensitivity to chemotherapeutics, providing a rationale for plasma cells to look for ways to suppress its expression to avoid cell death. We therefore first over-expressed wt PTPROt or an empty vector control, and then exposed stable cell lines to bortezomib over a range of concentrations for 24 hours. Interestingly, when normalized to vehicle-treated controls, both ANBL-6 (Figure 3A) and KAS-6/1 (Figure 3B) cells over-expressing wt PTPROt were consistently more sensitive to the induction of programmed cell death by bortezomib. Flow analysis of KAS-6/1 cells showed that this was associated with dual activation of Caspase 8 (Figure 3C, left panel) and Caspase 9 (Figure 3C, middle panel). Notably, this finding was not restricted to bortezomib, as ANBL-6 cells over-expressing wt PTPROt also had increased levels of Caspase cleavage after exposure to cisplatin (Figure 3D, left and right panels), and melphalan (Figure 3D, right panel), both of which are commonly used chemotherapeutics against myeloma.

### Decreased AKT activation in PTPROt over-expressing cells

**Figure 4:**
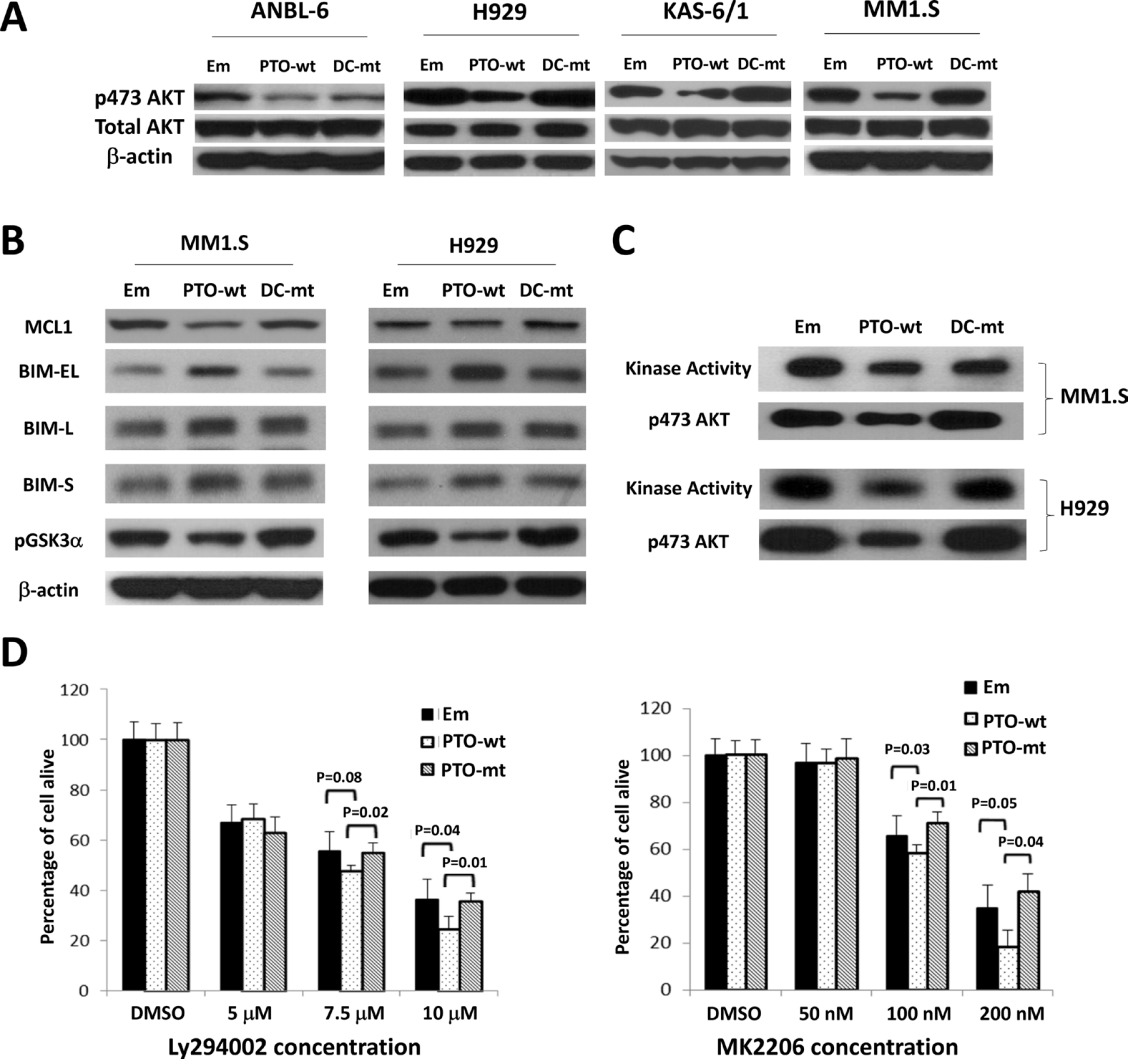
Impact of PTPROt on AKT signaling (**A***)* Lysates from ANBL-6, H929, KAS-6/1, and MM1.S cells expressing wt (PTO-wt) or mut *PTPROt* (DC-mut), or an empty vector control (Em), were analyzed by Western blotting for phospho-Ser473-AKT, total AKT, or β-actin. (**B**) The membranes with lysates from MM1.S and H929 cells were then re-examined for the expression and activation status of downstream AKT targets including MCL1, BIM isoforms, and phospho-Ser219-GSK. (**C**) An *in vitro* AKT kinase activity assay was performed using an antibody to precipitate AKT, followed by exposure of the precipitate to a GSK fusion protein in the presence of ATP. Western blotting was used to show AKT kinase activity (top panel for each cell line), as well as the level of phospho-AKT(S473) (bottom panels). (**D**) MM1.S cells expressing the indicated constructs were exposed to vehicle (DMSO), the PI3K inhibitor LY294002 (left panel), or the AKT inhibitor MK2206 (right panel). A tetrazolium assay was then used to determine viability with experiments performed in five replicates. Error bars represent standard deviation and statistical significance values are provided in the panels.

Protein kinase B/AKT signaling has been linked to bortezomib resistance [24–26], and we therefore wondered if PTPROt could influence AKT. To test this, we established MM1.S-, ANBL-6-, KAS-6/1-, and H929-derived cells containing an empty vector, or a wt or phosphatase-dead mutant PTPROt, and examined AKT phosphorylation [27]. Compared to the vector control, wt PTPROt reduced phospho-Ser473 levels in all four cell lines (Figure 4A). In contrast, the phosphatase-dead DC mutant did not have this effect in three cell lines, though there was some reduction in ANBL-6 cells, suggesting possible involvement of a phosphatase domain-independent component in suppression of AKT signaling by PTPROt in this one line. Downstream AKT targets include Myeloid cell leukemia-1 (MCL1)[28], BCL-2 interacting mediator of cell death (BIM)[29], and Glycogen synthase kinase-3 alpha (GSK3)[30]. When we looked at these, anti-apoptotic MCL1 was decreased in MM1.S and H929 cells (Figure 4B) in the presence of wt PTPROt. Conversely, pro-apoptotic BIM isoforms, including BIM_EL_, were increased, while inhibitory GSK3 phosphorylation was reduced, implying that AKT activity was reduced. Next, we performed AKT kinase assays using a GSK3 fusion protein, which confirmed that reduced substrate phosphorylation was seen in MM1.S and H929 cells over-expressing wt PTPROt, but not the empty vector or DC mutant (Figure 4C). These corresponded with lower levels of phospho-AKT in the presence of the wt PTPROt. Finally, MM1.S cells expressing wt or DC mutant PTPROt were treated with the Phospho-inositide 3-kinase (PI3K) inhibitor LY294002 [31], and the AKT inhibitor MK2206 [32]. While both drugs reduced cellular viability in a concentration-dependent manner, the presence of wt PTPROt potentiated this effect (Figure 4D), likely due to the lower level of AKT activity in these cells at baseline.

### PTPROt interacts with and dephosphorylates IQGAP1

**Figure 5:**
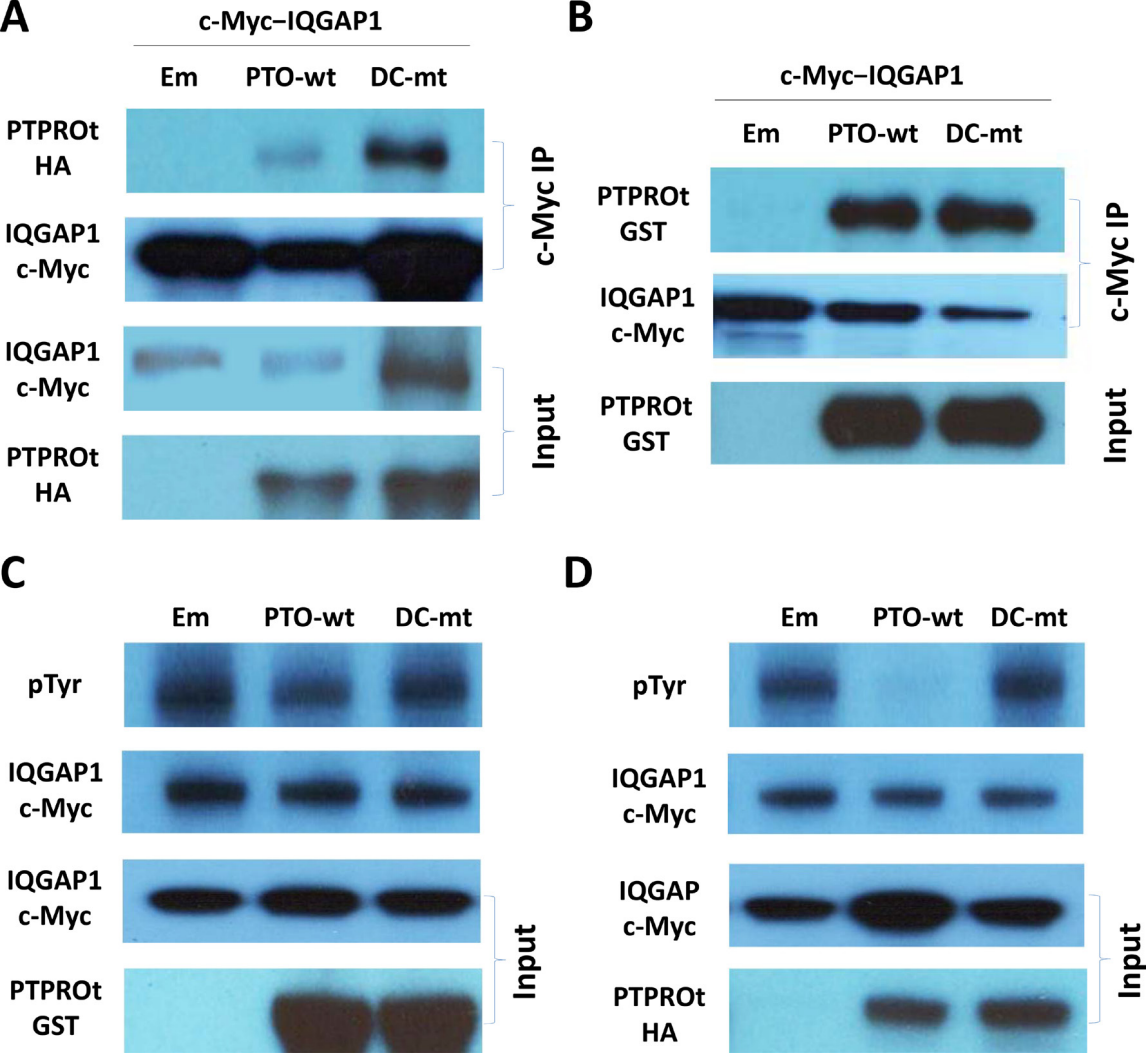
PTPROt interactions with IQGAP1 (**A)** Lysates from 293T cells co-transfected with vectors expressing c-Myc-tagged *IQGAP1* and either hemagglutinin (HA)-tagged wt *PTPROt*, DC-mt *PTPROt*, or an empty vector were subjected to immunoprecipitation with an anti-c-Myc antibody. Precipitates were then analyzed for the indicated proteins on Western blot using anti-c-Myc and anti-HA antibody. (**B**) Lysates from *E. coli* expressing GST-tagged *wt PTPROt*, DC-mt *PTPROt*, or an empty vector were subjected to pull down with c-Myc–tagged IQGAP1 (expressed and purified from 293T cells) and c-Myc antibody beads. Precipitates were then analyzed for the indicated proteins on Western blot using anti-c-Myc and anti-GST antibodies. (**C**) c-Myc-tagged IQGAP1 (expressed and purified from 293T cells) and GST-tagged wt PTPROt, DC-mt PTPROt, or empty vector (expressed and purified from *E. coli*) were combined and incubated in dephosphorylation buffer for one hour. Products were then analyzed for the total and phosphorylated IQGAP1 using anti-c-Myc tag and anti-pTyr antibodies, respectively. (**D**) c-Myc-tagged IQGAP1 (expressed and purified from 293T cells) and wt PTPROt, DC-mt PTPROt, or empty vector (expressed and purified from 293T cells) were combined, reacted and then analyzed as in *C*.

Since PTPROt is a phosphatase, the most straightforward mechanism for it to inhibit AKT would be through direct binding and dephosphorylation of this target. To examine if this could be the case, we over-expressed hemagglutinin (HA)- and FLAG-tagged wt or DC mutant PTPROt, as well as a vector control, and subjected the cells to tandem immunoprecipitation with anti-HA and anti-FLAG antibodies. The obtained, highly purified immunoprecipitates were then subjected to mass spectrometry analysis. One of the highest scoring peptides identified with both wild-type and substrate-trapping DC mutant PTPROt was IQ motif containing GTPase activating protein 1 (IQGAP1) (Table 1), a scaffold protein, which has been described as an activator of AKT signaling [33, 34]. In order to further substantiate the existence of a PTPROt-IQGAP1 interaction, we co-transfected 293T cells with vectors expressing c-Myc-tagged IQGAP1 and HA-tagged wt or DC mutant PTPROt. Immunoprecipitation was then performed from cell lysates with an anti-c-Myc antibody, and showed that both wt and DC mutant PTPROt were precipitated in concert with IQGAP1, although the co-precipitation of wtPTPROt was less efficient (Figure 5A). 293T expressed c-Myc-tagged IQGAP1 had also pulled down wt and DC mutant PTPROt expressed in *E. coli* (Figure 5B).

**Table 1:** PTPROt interacting partners identified by co-immunoprecipitation and mass spectrometry^1^

Gene Symbol	Wild-type PTPROt	Mutant PTPROt	Entrez Gene Name
PTPRO		+	Protein tyrosine phosphatase, receptor type, O
ADAD1		+	Adenosine deaminase domain containing 1 (testis-specific)
CACNA1I		+	Calcium channel, voltage-dependent, t type, alpha 1I subunit
GHSR		+	Growth hormone secretagogue receptor
HNRNPU		+	Heterogeneous nuclear ribonucleoprotein U (Scaffold attachment factor A)
ZNF638		+	Zinc finger protein 638
ADAMTS5	+		ADAM metallopeptidase with thrombospondin type 1 motif, 5
RPL13	+		Ribosomal protein L13
IQGAP1	+	+	IQ motif containing GTPase activating protein 1
LSM14A	+	+	LSM14A, SCD6 homolog A (S. cerevisiae)

^1^A “+” indicates detection of an interaction with either wild-type or substrate trapping DC mutant PTPROt or both.

In that IQGAP1 is a common substrate of receptor tyrosine kinases [35], we next hypothesized that a physical interaction between PTPROt and IQGAP1 could lead to dephosphorylation of the latter. We therefore performed an *in vitro* dephosphorylation assay using c-Myc-tagged IQGAP1 immunoprecipitated from 293T cells as the substrate, and GST-tagged wt or DC mutant PTPROt expressed in *E. coli* as the enzyme in the reaction. Confirming our assumption, the wt but not the substrate-trapping DC mutant PTPROt reduced the level of tyrosine phosphorylation of IQGAP1 (Figure 5C) compared to controls. We repeated these experiments with HA-tagged wt or DC mutant PTPROt expressed in 293T cells as the enzyme. Here, in an eukaryotic cell background, PTPROt almost quantitatively reduced IQGAP1 phosphorylation (Figure 5D), indicating that perhaps further post-translational modifications in 293T cells activate PTPROt to a greater extent than is possible in prokaryotic cells.

### Clinical relevance of PTPROt expression

**Figure 6:**
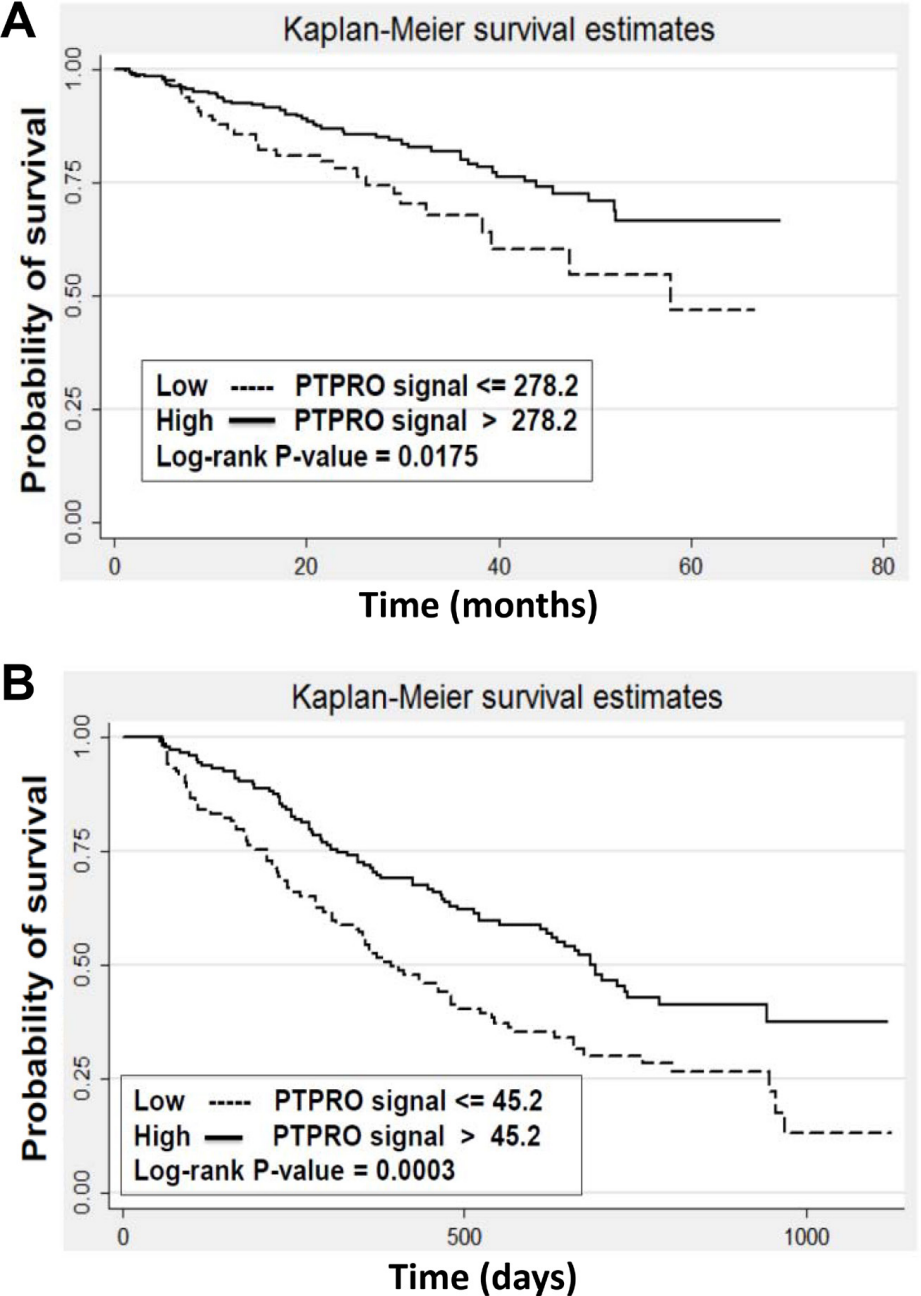
Expression of *PTPROt* and myeloma patient outcomes Kaplan-Meier survival analysis was performed from (**A**) the University of Arkansas dataset (*N* = 414) of newly diagnosed patients receiving bortezomib-based therapy, and (**B**) the Millennium Pharmaceuticals dataset (*N* = 264) of patients with relapsed/refractory myeloma who received single-agent bortezomib. Patients were stratified into groups with a greater than median *PTPROt* expression (solid line curves) or an expression that was at or below the median (dotted line curves). Log-rank *P*-values were calculated and are indicated in the two panels.

To explore the possibility that *PTPROt* could be of clinical relevance in predicting patient outcomes, we first analyzed the Millennium Pharmaceuticals dataset. This contains clinically annotated GEP data of myeloma patients with relapsed and/or refractory disease who were treated on phase II or III studies with bortezomib. Responses in this database were classified using the European Group for Blood and Marrow Transplantation criteria as fitting either a complete response (CR), partial response (PR), minimal response (MR), stable disease (SD), or progressive disease (PD). Patients were then stratified into high or low *PTPROt* expression groups based on the hybridization signals of the *PTPROt* probe from the cDNA microarray data as described in Materials and Methods. Notably, 9/14 (64%) patients who achieved a CR had high *PTPROt* expression levels (Table 2), while among patients who experienced PD, 45/66 (68%) had low *PTPROt* expression levels (*P* < 0.05). Differences between patients who achieved a CR or PD and any other response group were not statistically significant. We next analyzed the Arkansas (*N* = 414) and the Millennium Pharmaceuticals (*N* = 264) datasets from the Multiple Myeloma Genomics Portal (MMGP) (http://www.broadinstitute.org/mmgp/home) to examine if *PTPROt* could have an impact on long-term outcomes. In the Arkansas dataset of patients treated with a bortezomib-based induction regimen, those with a *PTPROt* expression level higher than the median had a superior overall survival than those whose myeloma cells had a median or lower *PTPROt* expression (Figure 6A; *P* = 0.0175). Similarly, in the Millennium dataset, higher *PTPROt* expression was associated with a better median survival (Figure 6B; *P* = 0.0003). Kaplan-Meier analysis of the latter showed that the median survival in the high *PTPROt* group was 691 days, while for the low group it was 391 days. Cox regression analysis (Breslow method) also showed that for every 100 interval of *PTPROt* hybridization signal the hazard ratio was 0.426587 (*P* = 0.0046).

**Table 2:** *PTPROt* expression levels and clinical responses to bortezomib from the Millennium Pharmaceuticals database on the Multiple Myeloma Genomics Portal

Response	PTPROt Expression Level		Proportion with High PTPROt (%)
	High	Low	
CR^*1^	9	5	64.3
PR	31	45	40.8
MR	11	12	47.8
NC	27	33	45.0
PD^*^	21	45	31.8

^*^P < 0.05

^1^Abbreviations: CR, complete response; MR, minor response, NC, no change; PD, progressive disease; PR, partial response.

## DISCUSSION

PTPRO is an R3 receptor-type protein tyrosine phosphatase (PTP) expressed in lymphoid cells as an alternatively spliced variant, PTPROt, with a short, 8 amino acid long, extracellular domain [22]. Available data suggest that *PTPROt* is developmentally regulated during B-cell differentiation, and may play a role as a tumor suppressor in lymphomagenesis. Its levels are decreased in germinal center-derived lymphomas, in which exogenous PTPROt expression induced G_0_/G_1_ cell cycle arrest, indicating its role in the growth control of B-cells [22]. This may occur in part through the ability of PTPROt to dephosphorylate the Spleen tyrosine kinase SYK, producing an effect on tonic B-cell receptor (BCR) signaling [23]. Interestingly, *PTPROt* may itself be a target for repression by BCL6 as the latter modulates tonic BCR signaling in diffuse large B-cell lymphoma [36]. However, its function in the context of multiple myeloma had not previously been studied. We found *PTPROt* expression was down-regulated in bortezomib-resistant models (Figure 1), while its over-expression suppressed myeloma proliferation and induced apoptosis (Figure 2). Moreover, rescue experiments in which *PTPROt* was expressed in bortezomib-resistant cells, and studies in drug-naïve cells, showed that it sensitized these models to proteasome inhibition, as well as the alkylating agent melphalan and the alkylating-like drug cisplatin (Figure 3). An association was then found between increased PTPROt expression and reduced levels of phospho-AKT, as well as reduced expression or activity of downstream AKT targets (Figure 4). Biochemical studies showed that PTPROt associated with and dephosphorylated IQGAP1 (Figure 5), providing one possible link between *PTPROt* and AKT. Finally, mining of clinical trial databases showed a direct relationship between increased *PTPROt* and both improved response quality to bortezomib (Table 2) as well as a longer overall survival after bortezomib-based therapy (Figure 6).

An interesting question which we are currently studying is the manner in which expression of *PTPROt* is down-regulated by plasma cells during the course of acquiring bortezomib resistance. One possibility is that *PTPROt* is known to be subject to aberrant methylation [37, 38], and in some malignancies, such as breast cancer, this has been associated with an inferior prognosis [39]. Notably, a recent study found aberrant methylation of several membrane-bound tyrosine phosphatase genes in acute lymphoblastic leukemia including *PTPRO*, and this was associated with a reduction in phosphorylated Extracellular signal-regulated kinases 1/2 and AKT, among others [40]. Another mechanism could be through loss of heterozygosity (LOH), and indeed LOH at the 12p12.3 locus of *PTPROt* has been identified in childhood acute lymphoblastic leukemia, where mapping studies suggested the presence of a tumor suppressor other than *p27*^KIP1^ [41]. Notably, in preliminary studies of array comparative genomic hybridization data from the MMGP, we found that 6/62 (9.7%) patients and 12/48 (25%) myeloma cell lines had decreased copy number of the 12p12.3 region (not shown), suggesting that loss of one *PTPRO* copy is a not infrequent occurrence in myeloma.

**Figure 7:**
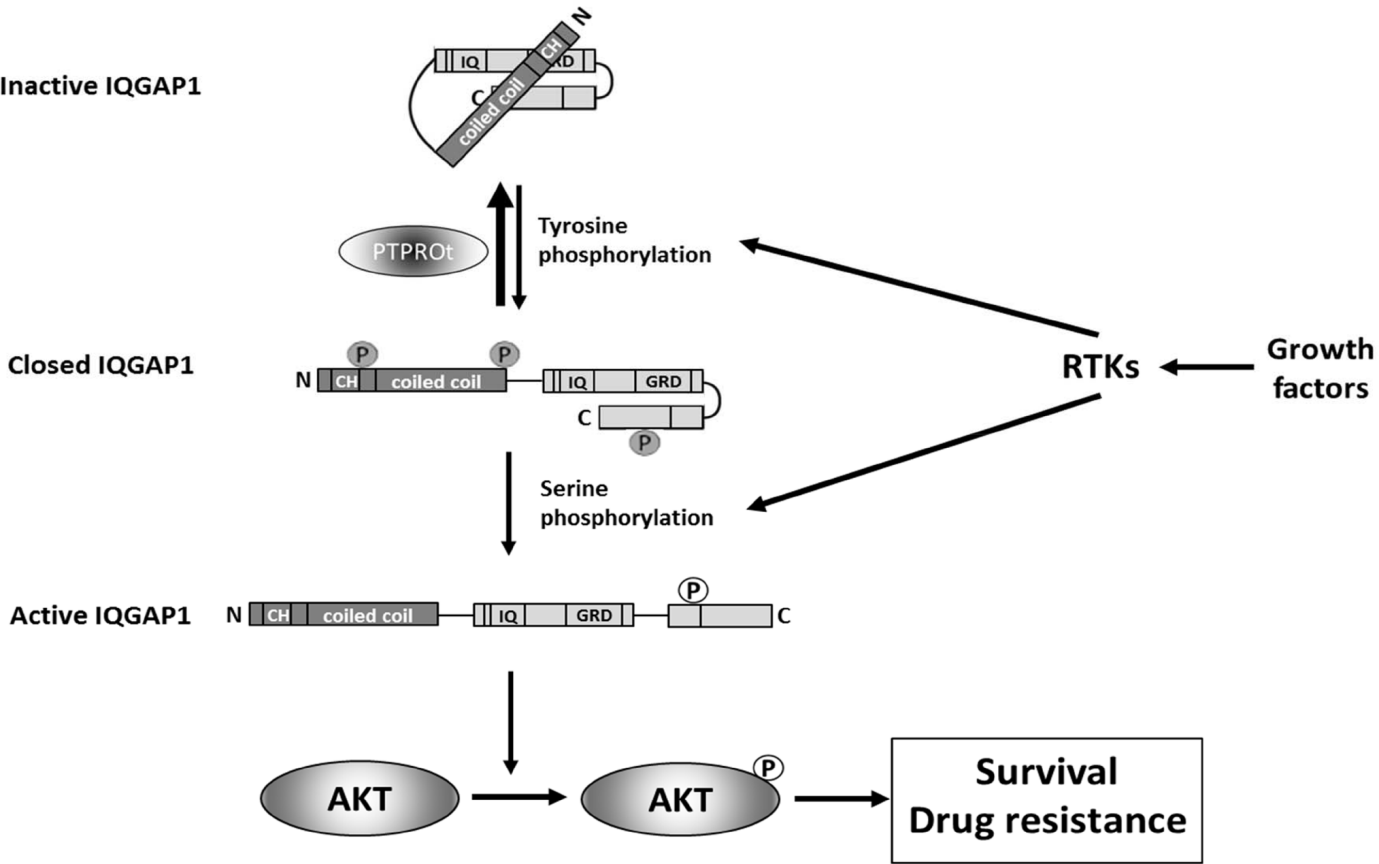
A model illustrating the role that PTPROt may play in modulating the IQGAP1 activation status Growth factor-mediated activation of receptor tyrosine kinases (RTKs) induces tyrosine phosphorylation of IQGAP1 [35, 49], that converts inactive IQGAP1 [50] to a closed IQGAP1 conformation [51]. Subsequent serine phosphorylation in response to activation of RTKs [52, 53] after opens C-terminus of IQGAP1 converting it into active form [54] that stimulates phosphorylation of AKT at serine 473 and, thus, activates AKT [52] resulting in pro-survival signaling and drug resistance. The initial tyrosine phosphorylation of IQGAP1 can be reversed by PTPROt-mediated de-phosphorylation (this report) interfering with activation of IQGAP1 and subsequently also AKT.

Our mechanistic data provide a novel pathway through which PTPROt may influence AKT signaling. A just published study noted that *PTPRO* -/- mice showed increased activation of AKT [42] but did not identify a possible mechanism. Another recent publication found that R3 receptor-like protein tyrosine phosphatase subfamily members inhibit insulin signaling by dephosphorylating the insulin receptor [43]. This could in part account for our finding that PTPROt reduces AKT activation given the prominent role of insulin signaling in regulating AKT activity [44]. However, we performed co-immunoprecipitation studies showing an association between PTPROt and IQGAP1, and *in vitro* dephosphorylation assays showing the latter was a substrate for the former. Moreover, we have also found that over-expression of IQGAP1 is by itself sufficient to enhance AKT activation in the absence of exogenous serum or growth factors (not shown). Thus, PTPROt appears to be able to regulate AKT through both an upstream impact on pathway activation, as well as through an IQGAP1-dependent mechanism by dephosphorylating the latter and placing it in an inactive conformation (Figure 7).

With regard to bortezomib resistance, our previous studies showed that this was mediated in part through an increase in Insulin-like growth factor 1 secretion by myeloma cells. This led to an increased autocrine and paracrine stimulation of AKT signaling which made myeloma cells more dependent on this pathway for survival, and therefore more sensitive to its blockade [21]. The current data provide another mechanism by which myeloma cells activate pro-survival AKT, and further underscore the importance that this pathway may have in proteasome inhibitor resistance. Moreover, as AKT activation is associated with resistance to other drug classes [45], *PTPROt* expression could influence the efficacy of other chemotherapeutics used against myeloma, as well as other malignancies. A number of AKT inhibitors are being evaluated in the clinic against relapsed/refractory myeloma, such as afuresertib, which has shown some activity as a single agent [46] and possibly the ability to overcome bortezomib resistance [47]. Our data suggest that *PTPROt* could be used as a biomarker to select patients who might most benefit from AKT inhibitors, since patients with low expression of this gene would be predicted to have increased AKT activation. Their disease could be more dependent on AKT signaling for survival and therefore more sensitive to AKT blockade as a strategy to achieve either chemosensitization, or to overcome proteasome inhibitor resistance. Prospective studies will be needed, however, to validate this hypothesis, though the finding that *PTPROt* expression levels are related to bortezomib sensitivity in existing datasets provide strong impetus for such trials. Since proteasome inhibitor resistance is an emerging clinical problem, these studies therefore provide a number of possible avenues for clinical translation of approaches that could restore sensitivity to this important class of agents.

## MATERIALS AND METHODS

### Cell culture and reagents

ANBL-6, KAS-6/1, and RPMI 8226, as well as bortezomib resistant (BR) counterparts of some of these cell lines were obtained, derived, and propagated as described previously [21]. MM1.S, H929, MOLP-8, and 293T cell lines were obtained from ATCC and cultured in FBS-supplemented (10%) RPMI 1640 and DMEM media, respectively. Bortezomib, cisplatin, and melphalan were purchased from Sigma-Aldrich (St. Louis, MO), MK2206 was purchased from Selleckchem (Houston, TX), and LY294002 was purchased from Cell Signaling Technologies (Danvers, MA).

### Gene expression profiling

GEP was obtained essentially as described previously [48]. Briefly, a biotinylated cRNA was generated from 300 ng of total RNA from wild-type and BR cells by the Eberwine procedure using the Illumina TotalPrep RNA Amplification kit (Invitrogen-Life Technologies; Grand Island, NY). Obtained cRNAs were hybridized overnight to Illumina HT-12 BeadArrays (Illumina; St. Diego, CA), stained with streptavidin-Cy3 (Invitrogen-Life Technologies), and scanned using a BeadArray Reader (Illumina).

### Real-time RT-PCR

Total cellular RNA was isolated using the TRIzol reagent (Invitrogen-Life Technologies). First-strand cDNA was synthesized using the High Capacity cDNA Reverse Transcription Kit (Applied Biosystems; Grand Island, NY) and subjected to real-time RT-PCR with made-to-order assays specific for PTPRO (ID, HS00243097_m1) or GAPDH (ID, Hs99999905_m1, endogenous control) and the TaqMan Universal PCR Master Mix (Applied Biosystems). Reactions were carried out in triplicate using the ABI PRISM 7900 HT Sequence Detection System (Invitrogen-Life Technologies), and the relative amount of product was determined by the comparative Ct method.

### Cell viability and apoptosis assays

The WST-1 tetrazolium reagent (Roche Diagnostics; Indianapolis, IN) was used to determine cellular viability according to the manufacturer’s instructions, typically with 1 × 10^4^ cells/well in 96-well plates. Cellular apoptosis was evaluated by flow cytometry after staining with Annexin V (BioVision; Mountain View, CA) and either TO-PRO-3 (Invitrogen-Life Technologies) or propidium iodide (PI). After the indicated drug treatments, cells seeded at 2 × 10^5^/well in 1 mL of growth medium in 24-well plates were collected, and washed with phosphate-buffered saline (PBS). Cells were then either resuspended in binding buffer and incubated as above at room temperature prior to flow cytometry, or they were fixed with 1% paraformaldehyde followed by incubation with 70% ethanol overnight prior to subsequent analysis. In some cases, treated cells were harvested for detection of activated Caspase 8 and 9 using the CaspGlow™ Red Active Caspase-8 and 9 Staining Kit from Biovision (Mountain View, CA) according to the manufacturer’s recommendations.

### Cloning and lentiviral transduction

*PTPROt* was PCR amplified from a cDNA clone (cat, SC128012, Origene; Rockville, MD) using the forward primer 5’-GGAATTCCACC ATGGTTACAGAGATGAATCCCAATG-3’, which included an EcoRI site and the reverse primer, 5’-GGGATCCGGCATAATCAGGAACATCATAAGGG TAGGACTTGCTAACATTCTCGTATATG-3’, which incorporated and hemagglutinin (HA) coding sequence and a BamHI site. PCR products were then cloned into the pCDH-MCS-T2A-copGFP-MSCV plasmid (System Biosciences; Mountain View, CA) through the same two enzyme sites. The resultant construct was called pCDH-MCS-PTOwt-HA-T2A-copGFP-MSCV. Finally, the DNA oligo CTAGCAATGG ACTACAAAGACGATGACGATAAAGCATACCCTTAT GATGTTCCTGATTATGCCGATTATAAGGATGACGAT GACAAGGCTGGG, which encoded two FLAG tags and one HA tag, was inserted into pCDH-MCS-PTOwt-HA-T2A-copGFP-MSCV through NheI and EcoRI sites. The QuikChange Site-directed Mutagenesis Kit (Stratagene; LaJolla, CA) was used to generate PTPROt substrate-trapping catalytic domain-inactivating mutants (PTPROT-CS [C325S], DA [D291A], and DC with mutated both sites). For IQGAP1 cDNA expression, the c-Myc-tagged IQGAP1 coding region was digested from pcDNA3-Myc-IQGAP1 (*#*30118, Addgene; Cambridge, MA) and sub-cloned into pLVX-DsRed-mono-C1 (Clontech Laboratories, Inc.; Mountain View, CA).

All recombinant Lentiviruses were produced by transient transfection of 293T cells according to standard protocols. Briefly, subconfluent 293T cells were co-transfected with 20 μg of an expression vector, 15 μg of pAX2, and 5 μg of pMD2G-VSVG by calcium phosphate precipitation. Medium was changed after 16 hours and recombinant Lentivirus vectors were harvested 24- and 48-hours later. Raw virus supernatants were concentrated by PEG precipitation, and target cells were transduced with a comparable amount of cDNA encoding Lentivirus vectors or corresponding empty vectors in growth medium containing 6 μg/mL polybrene. Transduced cells were subjected to drug selection or sorted by flow cytometry 5 days post-infection.

### Western blotting and antibodies

Cells were lysed in lysis buffer (20 mM Tris-HCl, pH 7.5, 150 mM NaCl, 1 mM Na_2_EDTA, 1 mM EGTA, 1% Triton, 2.5 mM sodium pyrophosphate, 1 mM β-glycerophosphate, 1 mM Na_3_VO_4_, 1 μg/mL leupeptin) containing complete Protease Inhibitor Cocktail tablets (Roche Diagnostics) and 1 mM phenylmethyl-sulfonyl-fluoride (PMSF). After three freeze-thaw cycles, lysate supernatants were obtained by centrifugation at 14,000 g for 15 minutes. Equal amounts (10–100 μg/lane) of protein were loaded on 10% SDS-PAGE gels and subjected to electrophoresis. Separated proteins were then transferred onto PVDF membranes and probed using mouse monoclonal anti-HA (Sigma-Aldrich), mouse monoclonal anti-AKT and rabbit monoclonal anti-phospho-AKT (Ser473), rabbit monoclonal anti-phospho-Glycogen synthase kinase (GSK)-3α/β (Ser219), anti-BIM, anti-MCL1, anti-cleaved Caspase 3, 8, or 9 (Cell Signaling Technology) antibodies, followed by a corresponding Horseradish peroxidase (HRP)–conjugated secondary antibody. The membranes were developed by enhanced chemiluminescence and exposed on Hyperfilm-ECL (both from GE Healthcare Biosciences; Pittsburgh, PA).

### Immunoprecipitation and mass spectrometry

MM1.S cells were transduced with Lentiviruses to express HA- and FLAG-tagged wild type (wt)-PTPROt, mutant DC-PTPROt, or empty vector. The cells were treated with 1 mM H_2_O_2_ and 1 mM Na_3_VO_4_ for 30 minutes, and then put in the lysis buffer (20 mM Tris-HCl, pH 7.5, 100 mM NaCl, 1 mM EDTA, 1% Triton X-100, 10% glycerol, 5 mM iodoacetic acid, and protease inhibitors) for 30 minutes on ice. Precipitation was performed using the FLAG HA Tandem Affinity Purification Kit (Sigma Aldrich) and the final eluted proteins were submitted for mass spectrometry analysis. Fragmentation spectra results were then searched against the SwissProt protein database with Mascot, and protein scores were derived from ions scores as a non-probabilistic basis for ranking protein hits. Ions scores were calculated as -10*Log(P), where P was the probability that the observed match was a random event, with individual ions scores >20 indicating identity or extensive homology (*p* < 0.05).

For the reverse immunoprecipitation, 293T cells were co-transfected with plasmids expressing c-Myc-tagged IQGAP1, and HA-tagged wt-PTPROt or DC-PTPROt, or empty vectors. The c-Myc-tagged IQGAP1 was purified from transfected 293T cells using c-Myc antibody and protein A/G magnetic beads (Pierce-Life Technologies) according to the manufacturer’s instructions. Purified protein and beads were then used to pull down Glutathione-S-transferase (GST)-tagged wt-PTPROt or DC-PTPROt expressed from pGEX-6p-1 in *E. coli*. Co-precipitated proteins were separated by SDS-PAGE gel electrophoresis and analyzed by Western blot as described above.

### *In vitro* AKT kinase assays

Assays were carried out using the AKT Kinase Assay Kit (Cell Signaling Technology) per the manufacturer’s instructions. Briefly, the cells were harvested and washed twice with PBS and subjected to lysis in the provided ice-cold buffer. Then, 250 μg of protein in 250 μl lysis buffer was immunoprecipitated with 20 μl of anti-phospho-AKT(S473) beads overnight at 4°C. After extensive washing, the immune-precipitates were incubated with 1 μg of GSK-3 fusion protein substrate in 50 μl of kinase buffer for 30 min at 30°C. Reactions were terminated by the addition of SDS loading buffer, samples were separated as described above, and substrate protein phosphorylation at Ser219 was detected by Western blotting.

### Dephosphorylation assay

To obtain a substrate for the assay, 293T cells were transfected with plasmid expressing c-Myc-tagged IQGAP1, and treated with 1 mM H_2_O_2_ and 1 mM Na_3_VO_4_ prior to the preparation of protein extracts in the presence of the phosphatase inhibitors. A highly phosphorylated c-Myc-tagged IQGAP1 substrate was then purified by immunoprecipitation using c-Myc antibody and protein A/G magnetic beads (Pierce-Life Technologies). GST fused wt PTPRO or DC mutant PTPRO were expressed from pGEX-6p-1 in *E. coli*. The inclusion bodies containing the expressed proteins were harvested, solubilized, and renatured using the Rapid GST Inclusion Body Solubilization and Renaturation Kit (Cell Biolabs, Inc.; San Diego, CA). The renatured proteins were purified using glutathione agarose according to the manufacturer’s instructions (Pierce-Life Technologies). HA-tagged wt or DC mutant PTPRO were also expressed in 293T cells and immunoprecipitated using EZview Red Anti-HA Affinity Gel according to the manufacturer’s instructions (Sigma-Aldrich). The IQGAP1 substrate was then reacted with wt PTPRO or DC-mutant PTPRO, purified either from *E. coli* or 293T cells, for 1 hour at 30°C in phosphatase buffer (25 mM HEPES, pH 7.4, 0.1 mM EDTA, 5 mM DTT, 50 mM NaCl). Reaction mixtures were then separated on SDS-PAGE gels and analyzed by Western blot using anti-c-Myc and -HA tag antibodies, and anti-phosphotyrosine antibody clone 4G10 (Millipore; Billerica, MA) was used to probe the phosphorylation status of IQGAP1.

### Data mining and statistical analysis

Three datasets were downloaded from the open-access Multiple Myeloma Genomics Portal (MMGP): the Arkansas dataset, which contains GEP data from CD138-selected plasma cells from 414 myeloma patient bone marrows with associated survival and classification data; the Millennium Pharmaceuticals dataset, which contains GEP data from purified myeloma samples of 264 patients enrolled in clinical trials of bortezomib, along with survival data, treatment, and response; and the Mayo Clinic Cell Line and Patient dataset, which contains the array comparative genomic hybridization (aCGH; Agilent 44k comparative genomic hybridization) from 46 myeloma cell lines and tumor samples from 62 myeloma patients. Kaplan-Meier survival curves and the Cox proportional hazard model from STATA were used for the analysis of correlation between PTPROt expression levels from GEP data sets and patient survival. Hybridization signals from probe set 208121_s_at, which is the only validated probe for PTPRO, were transformed into log10 values and stratified into high and low level groups if the log10 signal was more and equal or less than the average value, respectively, before they were put into the models. Patients’ responses in the Millenium dataset were classified using European Group for Bone Marrow Transplantation criteria as complete response (CR), partial response (PR), minimal response (MR), no change (NC), or progressive disease (PD). Differences between treatment response groups stratified by the GEP expression levels of *PTPROt* were calculated using Pearson’s Chi-squared test and *P* < 0.05 was considered statistically significant.

## Abbreviations

The abbreviations used are:

aCGH: array comparative genomic hybridization
BCR: B-cell receptor
BIM: BCL-2 interacting mediator of cell death
BR: bortezomib resistant
CR: complete response
GEP: gene expression profiling
GSK: glycogen synthase kinase
GST: Glutathione-S-transferase
HA: hemagglutinin
HRP: horseradish peroxidase
IGV: Integrative Genomics Viewer
IQGAP1: IQ motif containing GTPase activating protein 1
LOH: loss of heterozygosity
MCL1: Myeloid cell leukemia 1
MMGP: Multiple Myeloma Genomics Portal
MR: minimal response
mut: mutant
NC: no change
PBS: phosphate-buffered saline
PD: progressive disease
PI: propidium iodide
PMSF: phenylmethyl-sulfonyl-fluoride
PR: partial response
PTP: protein tyrosine phosphatase
PTPRO: Protein tyrosine phosphatase receptor-type O
PTPROt: truncated PTPRO
wt: wild-type
